# Case report: Response to everolimus in a patient with platinum resistant, high grade serous ovarian carcinoma with biallelic *TSC2* inactivation

**DOI:** 10.3389/fonc.2024.1357980

**Published:** 2024-03-27

**Authors:** Mariko Peterson, David L. Kolin, Panagiotis A. Konstantinopoulos

**Affiliations:** ^1^ Department of Pathology, Brigham and Women’s Hospital, Boston, MA, United States; ^2^ Department of Medical Oncology, Dana Farber Cancer Institute, Boston, MA, United States

**Keywords:** platinum resistant ovarian cancer, mTOR inhibitor, TSC1 and TSC2 mutations, everolimus, letrozole

## Abstract

**Background:**

Patients with platinum-resistant recurrent high grade serous ovarian carcinoma have poor outcomes and limited treatment options.

**Case presentation:**

We present a case of a 48-year-old woman with platinum-resistant high grade serous ovarian carcinoma harboring the pathogenic *TSC2* R611Q variant with concomitant single copy loss of *TSC2* (suggesting biallelic *TSC2* inactivation) identified in targeted tumor sequencing. The patient was treated with the mTOR inhibitor everolimus, with an excellent response by imaging and a marked decrease in CA125; she remained on everolimus for 19 months until she developed progressive disease.

**Conclusions:**

While mTOR inhibition is frequently used in tumors associated with tuberous sclerosis complex (TSC), such as lymphangioleiomyomatosis and malignant perivascular epithelioid cell tumors, this is the first case of a patient with ovarian cancer harboring *TSC1/2* mutations who responded to mTOR inhibition. This case highlights the utility of targeted DNA sequencing in the management of ovarian carcinoma and demonstrates the value of tumor-agnostic targeted therapies.

## Introduction

High grade serous carcinoma of the ovary (HGSC) is the most common ovarian malignancy, and usually presents with advanced stage disease (1). Genomically, virtually all HGSC are characterized by mutations in *TP53* and chromosomal instability, and approximately half are homologous recombination deficient (HRD), secondary to mutations or methylation of *BRCA1/2* or other genes in the homologous recombination pathway that render them susceptible to poly-ADP-ribose polymerase (PARP) inhibitors (2, 3). The mainstay of treatment is tumor debulking and carboplatin/paclitaxel chemotherapy, followed by maintenance therapy with bevacizumab and/or PARP inhibitors based on tumors’ HRD status. CA125 represents a reference marker for follow up and monitoring response to therapy in this disease and is approved by the US Food and Drug Administration (FDA) in that setting (4). Nearly 75% of patients with HGSC recur, and their disease eventually becomes resistant or refractory to platinum based chemotherapy. Treatment options for platinum resistant ovarian cancer consist of single-agent chemotherapy (which has limited activity), chemotherapy plus bevacizumab and more recently the antibody–drug conjugate (ADC) mirvetuximab soravtansine for tumors with high folate receptor α (FRα)-expression (~35-40% of all cases) (5). Management of platinum-resistant ovarian cancer remains a significant unmet medical need.

The genes *TSC1* and *TSC2* encode proteins which form the TSC1-TSC2 complex, which is a key negative regulator of mammalian target of rapamycin complex 1 (mTORC1) (6). Germline mutations in *TSC1/2* give rise to tuberous sclerosis complex (TSC), an autosomal dominant inherited disease, whose manifestations include facial angiofibromas, subependymal giant cell tumors, cardiac rhabdomyomas, renal angiomyolipomas, and lymphangioleiomyomatosis (LAM) (7). Angiomyolipomas and LAM are included in the group of tumors known as perivascular epithelioid cell tumors (PEComa), which are characterized by positivity for both smooth muscle and melanocytic immunohistochemical markers. Ovarian carcinomas are not a feature of TSC.

Tumors typically associated with TSC and which harbor mutations in *TSC1/2*, such as PEComa (8) and LAM (9) are responsive to mTOR inhibition. In the gynecologic tract, malignant uterine PEComas are often treated with mTOR inhibitors (10). However, *TSC1/2* mutations are also found in other tumor types. For example, rare uterine sarcomas harbor both *JAZF1::SUZ12* gene fusions and *TSC2* mutations, and these patients may respond to mTOR inhibition (11). Herein, we describe a patient with *TSC2*-mutated HGSC treated with everolimus.

## Case presentation

A 48-year-old G4P4 Caucasian lady with a history of papillary thyroid carcinoma presented for a routine umbilical hernia repair in 2016. She had positive family history for breast and prostate cancer on the paternal side, was never a smoker and underwent genetic testing for hereditary cancer syndromes which was negative. Intraoperatively, she was found to have a left hydrosalpinx and ovarian mass. A subsequent transvaginal ultrasound revealed a 10.9 cm solid-cystic left adnexal mass, and a CT abdomen/pelvis identified a liver lesion concerning for metastasis. A preoperative CA125 was 386 units/mL.

The patient underwent total abdominal hysterectomy, bilateral salpingo-oophorectomy, and tumor debulking. Pathologic examination showed HGSC involving the bilateral ovaries, left paratubal soft tissue, uterine serosa, pelvic peritoneum, left ureter, rectosigmoid colon, and diaphragm. The omentum was negative for tumor. Her stage was pT3b pNx pMx (FIGO IIIB).

**Figure 1:**
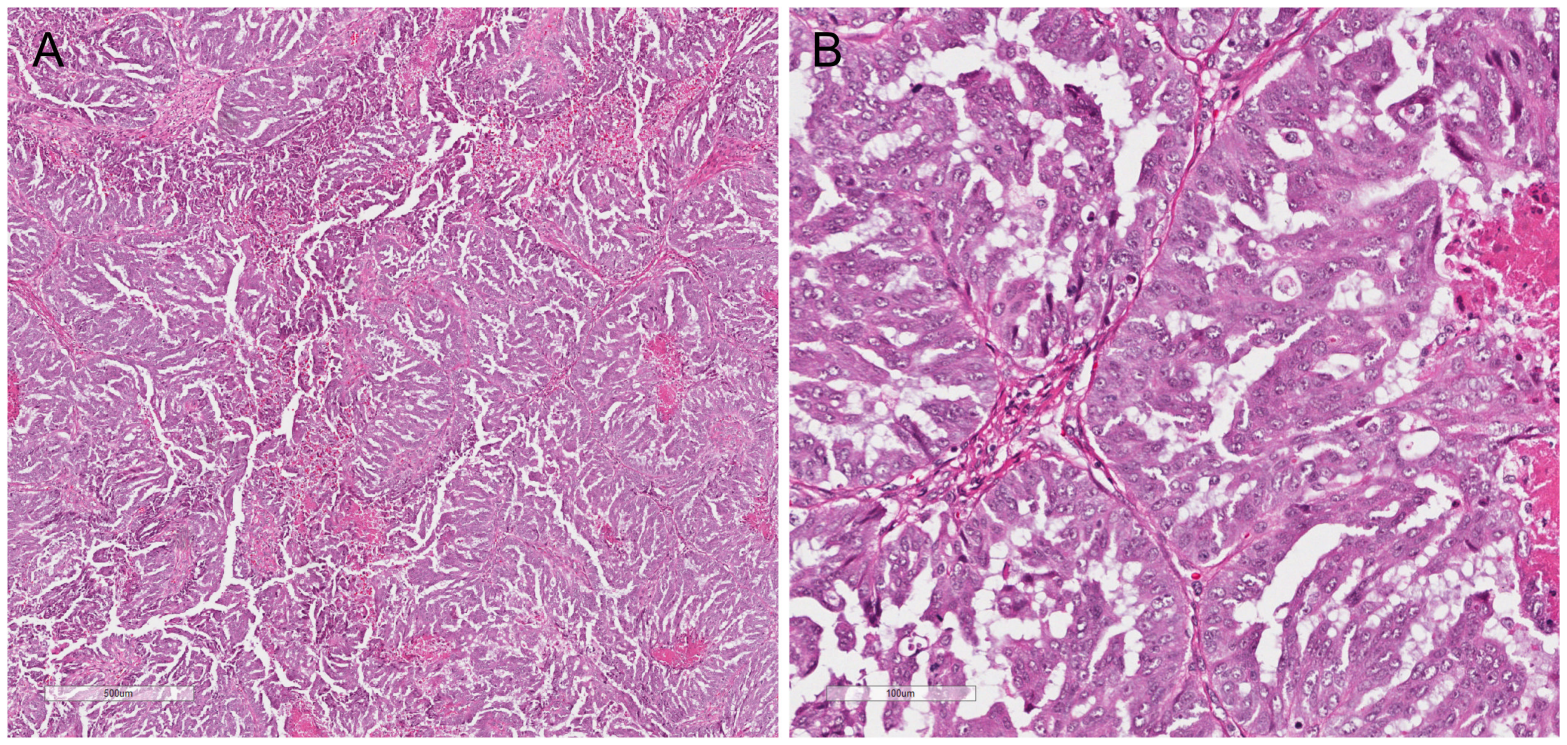
**(A)** High grade serous carcinoma with *TSC2* mutation, showing predominant gland formation with slit-like spaces (4X). **(B)** Cells show intermediate grade nuclear atypia, prominent nucleoli, and moderate eosinophilic cytoplasm (20X).

Microscopic examination revealed a carcinoma which predominantly formed glands and slit-like spaces (**Figure 1**). The cells showed moderate atypia and eosinophilic cytoplasm. Immunohistochemical studies demonstrated the tumor cells were positive for PAX8, WT-1, and folate receptor alpha (75%), and negative for HER2 (0).

Molecular profiling of the tumor was completed using the Oncopanel assay, a targeted next generation sequencing panel of 447 oncogenes and tumor suppressors, as previously described (12). The tumor harbored single nucleotide variants in *TSC2* c.1832G>A (p.R611Q), *TP53* c.587G>C (p.R196P), *BCL11B* c.2224G>C (p.E742Q), *BUB1B* c.1478C>T (p.T493I), *CBFA2T3* c.770C>T (p.T257M), *NSD1* c.708G>C (p.Q236H), *SETBP1* c.3712G>A (p.D1238N), *STK11* c.1263C>T (p.S421S), and *ZNRF3* c.334C>A (p.Q112K). Numerous copy number changes were identified, including single copy deletions involving *TP53* and *TSC2*. A *UBE4B::KIF1B* fusion, of uncertain biological significance, was also detected. A germline testing panel, which included *BRCA1*, *BRCA2*, *TSC1*, *TSC2*, and *STK11*, revealed no mutations.

**Figure 2:**
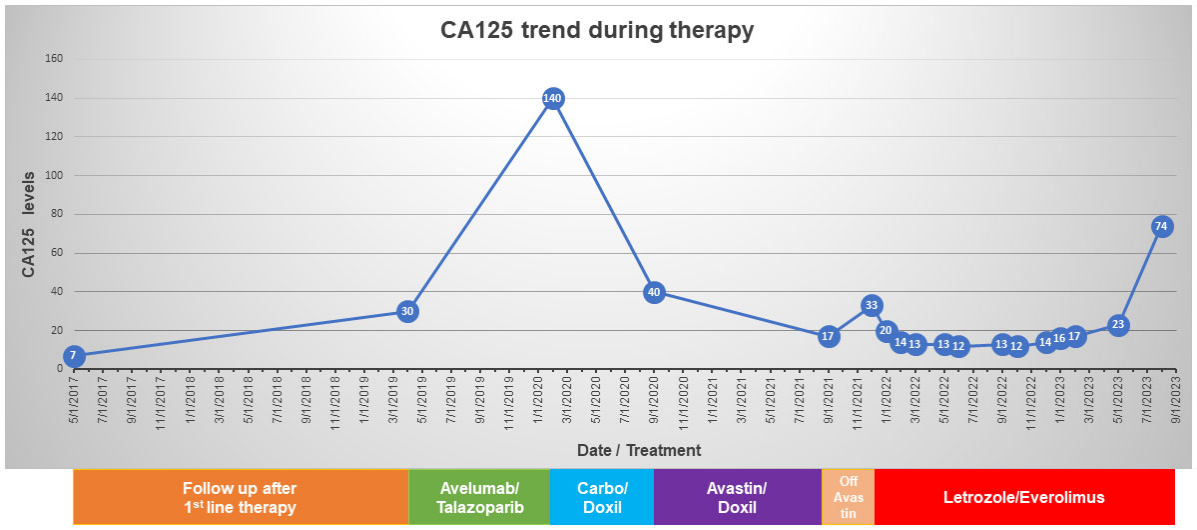
Timeline of CA125 levels and the treatments received by the patient.

Post-operatively, she underwent six cycles of intravenous and intraperitoneal paclitaxel and cisplatin to which she had a complete clinical response. Two and a half years after her initial surgery, a rising CA125 prompted imaging which revealed peritoneal and abdominal wall nodules. A biopsy confirmed recurrent HGSC. She was then enrolled in a clinical trial of PARP inhibitor talazoparib and anti-PD-L1 antibody avelumab. The patient was initially stable on this treatment, but after approximately one year, demonstrated radiographic and biochemical increases in disease burden. At that time, she transitioned to carboplatin and liposomal doxorubicin with good response, but she recurred again and was treated with bevacizumab and liposomal doxorubicin for her then platinum resistant disease. Her course was complicated by bevacizumab-induced hypertension, recurrent mucositis, and eventually renal toxicity, which prompted suspension of this regimen, during which her CA125 rose and she experienced recurrence of her peritoneal nodule which caused malignant hydronephrosis. She was subsequently started on everolimus 10mg po daily and letrozole 2.5mg po daily, due to the presence of the *TSC2* mutation with concomitant *TSC2* single copy loss. The everolimus dosing frequency was reduced to 5 days on/2 days off secondary to thrombocytopenia. CT scans 12 and 16 months after starting everolimus revealed a decreased tumor burden and a normal CA125 (16 and 23 units/mL respectively). Overall, she tolerated letrozole/everolimus well with preservation of her quality of life, i.e., she continued working and maintained all her previous activities. However, 19 months after initiation of everolimus, her CA125 increased to 74 units/mL and a CT scan showed worsening peritoneal carcinomatosis prompting discontinuation of everolimus (**Figure 2**).

## Discussion

In this report, we describe a patient with *TSC2*-mutant, platinum resistant HGS ovarian carcinoma who responded to everolimus. The combination of the point mutation and the single copy deletion of *TSC2* present in this tumor is suggestive of biallelic *TSC2* inactivation which has been associated with response to targeted mTOR inhibition. In the gynecologic tract, mTOR inhibitors have been used with success in both uterine PEComas and stromal sarcomas with *TSC2* mutations (10, 11). However, to our knowledge, this report is the first which describes response to mTOR inhibition in a *TSC2*-mutant ovarian carcinoma. This is an area of active interest in multiple tumor sites, and there is currently an ongoing phase II basket trial of nab-sirolimus for patients with *TSC1/2* mutated solid tumors (NCT05103358) (13). A prior phase II trial failed to identify a significant benefit to adding everolimus to bevacizumab in recurrent ovarian cancer (14); however, that study did not screen patients for alterations in the mTOR pathway. Furthermore, a phase 1 trial to determine the safety and tolerability of everolimus in combination with PARP inhibitor (PARPi) niraparib in patients with advanced ovarian or breast cancer was previously reported. This study demonstrated that the combination of everolimus and niraparib was associated with significant toxicity, even at lower doses of both agents, and was therefore not feasible; no *TSC2* mutations were identified in any of the patients in that study (15).

Among 316 HGSC in the ovarian cancer genome atlas analysis (2), 5 (1.6%) had alterations in *TSC1* (3 with a deep deletion, 1 with a truncating mutation and shallow deletion, and 1 with a missense mutation) and 3 (1%) had deleterious alterations in *TSC2* (1 with a deep deletion, and 2 with both a missense mutation and shallow deletion). So, while *TSC1/2* alterations appear uncommon in HGSC, a small proportion of cases have mTOR pathway aberrations and may benefit from targeted therapy. It is unclear if these tumors show distinct morphologic or immunohistochemical features, such as positivity for melanocytic markers as seen in PEComa. However, given how uncommon these mutations are in HGSC, sequencing with a targeted panel is likely the most effective way to identify patients who may benefit from consideration of mTOR inhibition.

The *TSC2* R611Q is a well characterized pathogenic variant that has been reported in multiple patients with TSC, an autosomal dominant inherited disease due to germline inactivating *TSC1/2* mutations (7, 16). Functionally, this amino acid substitution involves the TSC1 interaction domain of the TSC2 protein (tuberin) that leads to decreased interaction of TSC2 with TSC1 (hamartin) and abrogation of the TSC complex-dependent inhibition of mTORC1 activity (16). Constitutive activation of mTORC1 can be targeted via everolimus, a derivative of rapamycin, which forms a complex with FKPB12 that functions as an allosteric inhibitor of mTORC1. Accordingly, everolimus is active against multiple neoplasms occurring in patients with Tuberous Sclerosis Complex, including renal angiomyolipoma, lymphangioleiomyomatosis (LAM), and subependymal giant cell astrocytoma (9–11, 17).

It is important to underscore that while everolimus was chosen due to the biallelic *TSC2* inactivation, we elected to add hormonal therapy (using the aromatase inhibitor letrozole) to everolimus for two reasons. First, although HGSCs are not considered hormonally driven and are less responsive to hormonal therapy, weak or strong ER expression (≥1%) is observed in at least 80% of HGSC (18), and two studies (19, 20) have demonstrated that treatment with either tamoxifen or letrozole (both at first and at later recurrences) has activity against these tumors. Second, given the extensive crosstalk between the ER and the PI3K/mTOR pathways, synergistic antitumor activity has been demonstrated with combined hormonal therapy and mTOR inhibition, and the everolimus plus exemestane combination is currently approved by the FDA for the treatment of hormone receptor positive, human epidermal growth factor receptor 2 (HER2)–negative metastatic breast cancer after failure of treatment with letrozole or anastrozole (21). Of note, the combination of everolimus and letrozole has been studied in phase II trials in both the ovary (22) and endometrium (23); however, neither of these trials screened tumors for *TSC1/2* mutations to identify patients who might be particularly sensitive to this combination.

Given that activation of the PI3K pathway (which occurs as a result of *TSC2* inactivation) is a well-documented and established mechanism of resistance to hormonal therapy (24), *TSC2* mutations are expected to confer resistance, not sensitivity to letrozole. Nonetheless, we cannot possibly exclude that a component of this patient’s response to letrozole/everolimus may have been due to the letrozole given that hormonal therapy with tamoxifen or letrozole has demonstrated activity against HGSOC (19, 20), including at later recurrences (which was also the reason why letrozole was added to everolimus in this patient).

Eventually, this patient developed resistance to everolimus after 19 months of therapy, which is remarkable in the setting of platinum resistant HGS ovarian cancer. Key mechanisms of resistance to everolimus include the development of mTORC1 mutations (which may be overcome by drugs that target the ATP binding site of mTOR resulting in abrogation of its kinase activity) as well as feedback activation of the PI3K signaling pathway and paradoxical activation of MAPK/ERK signaling. Unfortunately, no biopsy after progression on everolimus was available for this patient, so no functional studies to determine the exact mechanism of acquired resistance to everolimus could be performed.

Moving forward, this patient would be an excellent candidate for the ADC mirvetuximab (which was not yet FDA approved at the time everolimus was initiated) given the high FRa expression in her tumor (defined as ≥75% positive cells with ≥2+ staining intensity using the percent stain 2 positive (PS2+) method). Specifically, in the SORAYA trial, mirvetuximab exhibited an ORR of 32.4% with a median duration of response of 6.9 months in patients with platinum-resistant ovarian cancer and high FRα expression by the PS2+ method (25). Furthermore, the confirmatory randomized phase 3 trial (MIRASOL) of mirvetuximab versus the investigator’s choice of chemotherapy in platinum-resistant FRα-high OC demonstrated statistically significant benefits for mirvetuximab in both median PFS and OS, the first therapy to demonstrate an OS advantage in platinum-resistant OC (26).

## Data availability statement

The original contributions presented in the study are included in the article/supplementary material. Further inquiries can be directed to the corresponding authors.

## Ethics statement

Ethical approval was not required for the study involving humans in accordance with the local legislation and institutional requirements. Written informed consent to participate in this study was not required from the participants or the participants’ legal guardians/next of kin in accordance with the national legislation and the institutional requirements. Written informed consent was obtained from the individual(s) for the publication of any potentially identifiable images or data included in this article.

## Author contributions

MP: Formal analysis, Writing – original draft, Writing – review & editing. DK: Formal analysis, Writing – original draft, Writing – review & editing. PK: Conceptualization, Formal analysis, Writing – original draft, Writing – review & editing.
